# Delayed endoscopic removal of sharp foreign body in the esophagus increased clinical complications: An experience from multiple centers in China

**DOI:** 10.1097/MD.0000000000016146

**Published:** 2019-06-28

**Authors:** Jingjing Yuan, Mengjie Ma, Yang Guo, Bili He, Zhenzhai Cai, Bin Ye, Lei Xu, Jiang Liu, Jin Ding, Zhongfa Zheng, Jianhua Duan, Liangjing Wang

**Affiliations:** a Department of Gastroenterology, Second Affiliated Hospital of Zhejiang University School of Medicine; b Institute of Gastroenterology, Zhejiang University, Hangzhou; c Department of Gastroenterology, Taizhou Hospital of Zhejiang Province, Taizhou; d Department of Gastroenterology, Second Affiliated Hospital of Wenzhou Medical University, Wenzhou; e Department of Gastroenterology, Central Hospital of Lishui City, Lishui; f Department of Gastroenterology, Ningbo First Hospital, Ningbo; g Department of Gastroenterology, Central Hospital of Huzhou City, Huzhou; h Department of Gastroenterology, Central Hospital of Jinhua City, Jinhua; i Department of Gastroenterology, Kecheng Hospital of Quzhou City, Quzhou; j Department of Gastroenterology, Second Hospital of Shaoxing City, Shaoxing, Zhejiang Province, China.

**Keywords:** complications, endoscopy, esophagus, foreign bodies

## Abstract

Foreign bodies impaction in the esophagus is a common clinical emergency. The aim of this study was to investigate the clinical features of foreign body ingestion, and to analyze the risk factors of complications during the endoscopic procedure.

From 18 general hospitals in Zhejiang Province in China, 595 patients who underwent gastroscopic removal of ingested foreign bodies were prospectively recruited. Patient characteristics, clinical features, foreign body features, clinical outcomes, and complications were documented.

The most common types of foreign body in the esophagus were sharp objects (75.9%), including fish bones (34.0%), chicken bones (22.1%), and fruit nuclei (17.1%). The remaining types were non-sharp objects (24.1%), including food bolus (14.6%). Most objects were lodged in the proximal esophagus (75.9%). Foreign body-related complications occurred in 63 patients (10.5%), including hemorrhage (5.0%), perforation and infection (5.5%). The complication rate was increased by 4.04- and 8.48- fold when endoscopic retrieval was performed after impacted for over 24 and 48 hours, respectively, after impaction, as compared with within 12 hours. Logistic regression analysis revealed that the patients with sharp objects developed more complications than those with non-sharp ones (odds ratio, 2.85; 95% confidence interval, 1.08–7.50; *P* = .034). However, complications were unrelated with the location in the esophagus or length of foreign bodies (*P* > .05).

Sharp objects were the most frequently ingested foreign bodies in the esophagus in China. The prevalence of complications was increased in the patients with long foreign body retention time (>24 hours) and sharp objects. Sharp foreign bodies in the esophagus are recommended to be removed within 24 hours.

## Abstract 2

Supplemental Digital Content is available in the text

## 1 Introduction

Foreign body (FB) ingestion is a common medical emergency accounting for 4% of all emergency endoscopies, secondary to the gastrointestinal (GI) bleeding.^[1]^ In adults, the most common FB is food bolus in Western world.^[1,2]^ However, in Asian countries, sharp FB including fish bones, chicken bones, fruit nuclei and dentures are the most common ingested objects.^[3,4]^ Most impacted FB can pass through the GI tract spontaneously. However, 10% to 20% still need clinical intervention, mostly endoscopic management, while less than 1% even require surgery.^[5]^ The successful removal rate by endoscopy could reach up to 95%.^[1,3,4,6]^ According to the latest guidelines and consensus established by the American Society for Gastrointestinal Endoscopy (ASGE),^[5]^ the North American Society for Pediatric Gastroenterology, Hepatology and Nutrition (NASPGHAN),^[7]^ and the European Society of Gastrointestinal Endoscopy (ESGE),^[8]^ the primary clinical treatment for ingested FBs is endoscopic management.

Under endoscopy, the most common local lesions include mucosal edema, erosion, laceration, ulcer, and oozing. FB-related complications comprise hemorrhage, perforation, obstruction, retropharyngeal or mediastinal abscess formation, and FB migration into facial spaces of the neck.^[1,3,9]^ Among complications, 3% to 20% were reported to be caused by FB ingestion.^[9,10]^ The type and location of FBs, and duration of impaction were correlated with the occurrence of complications.^[10–12]^ Specifically, sharp objects could increase the risk of perforation.^[13,14]^

For symptomatic patients, endoscopy has been regarded as the primary tool for removing impacted FB in the esophagus. According to the latest guidelines of the ASGE and ESGE,^[5,8]^ emergent endoscopy is recommended for impacted sharp-pointed objects within 24 hours, although the application of endoscopy for impacted food bolus is still controversial. Intravenous sedation is employed for the successful removal; however, it might not always be available because of the emergent situation. As the types of impacted FB differ between Asian and Western countries, the management approaches vary largely under different clinical conditions. This prospective study aimed to prospectively investigate the clinical features of FB ingestion and endoscopic removal in the esophagus, and to summarize the related risk factors of endoscopic complications in multiple endoscopy centers in China.

## 2 Methods

### 2.1 Patients

Patients with suspected FB ingestion in the esophagus who signed the consent forms were recruited between October 2015 and August 2016 in 18 tertiary hospitals in Zhejiang Province in East China. By using a uniform questionnaire, data on demographic and clinical variables including age, sex, past medical history, and clinical symptoms, were collected.

### 2.2 Endoscopic procedure and foreign bodies

All endoscopic management procedures were conducted by experienced specialists. Every endoscopist has more than 5 years’ experiences in diagnosis and treatment of digestive system diseases and finished the esophagogastroduodenoscopy independently more than 200 cases. During the procedure, impacted FBs were removed using accessory devices including rat-tooth forceps (FG-47L-1, Olympus, Japan), polypectomy snares (SD-6L-1, Olympus, Japan; REF-6031, Boston, USA), and dormier baskets (FG301-Q, Olympus, Japan; MWB-2X4, COOK, USA). All the patients were sedated with general anesthesia using 50 to 100 μg fentanyl and 1 to 2 mg/kg propofol or local anesthesia using 2% lidocaine mucilage.

After FB removal, macroscopic characteristics, reports including esophageal mucosal lesions, were recorded. Specifically, the features and locations of FBs, and the complications were described. Normally, FBs shorter than 2.5 cm could pass through the whole digestive tract,^[15]^ so we defined FBs less than 2.5 cm as short, and those longer than 6.0 cm as long in accordance with the guidelines or consensus of the ASGE and ESGE.^[5,8]^

### 2.3 Statistical analyses

Data were analyzed using STATA Version 13.0 (Stata Corp: College Station, TX). The Pearson chi-square or Fisher exact tests were used for categorical and ordinal variables. To summarize the independent predictive factors for related complications, multivariate analysis was performed with a logistic regression analysis. The odds ratio (OR) was calculated to assess the risk of the related factors. A *P* value of less than .05 was considered statistically significant.

## 3 Results

### 3.1 Patient characteristics

We enrolled 595 patients with suspected FB ingestion who were admitted to the endoscopic centers of 18 tertiary hospitals. Of the patients, 89 underwent a laryngoscopy first on the basis of the obvious throat pain complaints or positive imaging examination that suggested upper FB existence. FBs were found on laryngoscopy in only 9 patients, and were failed to remove; thus, another endoscopic management procedure was required. Among all the suspected patients, 561 had FBs visible under endoscopy. Underlying upper gastrointestinal tract diseases could affect the physiological function of the esophagus. In our study, 89 patients (15.0%) had GI diseases during endoscopic examination, including 52 with esophageal carcinoma, 18 with benign esophageal stricture, 12 with reflux esophagitis, and 5 with hiatal hernia (Table 1). Of the 595 patients, 573 patients (96.3%) had obvious symptoms after FB ingestion, of which approximately 10% had more than one symptom. Among these, 383 patients had odynophagia (64.4%), 262 had dysphagia (44.0%), and 91 had chest pain (15.3%). Other less common symptoms included nausea and vomiting (7 cases, 1.2%), abdominal pain (5 cases, 0.8%), and hematemesis (4 cases, 0.7%) (Fig. S1).

**Table 1:**
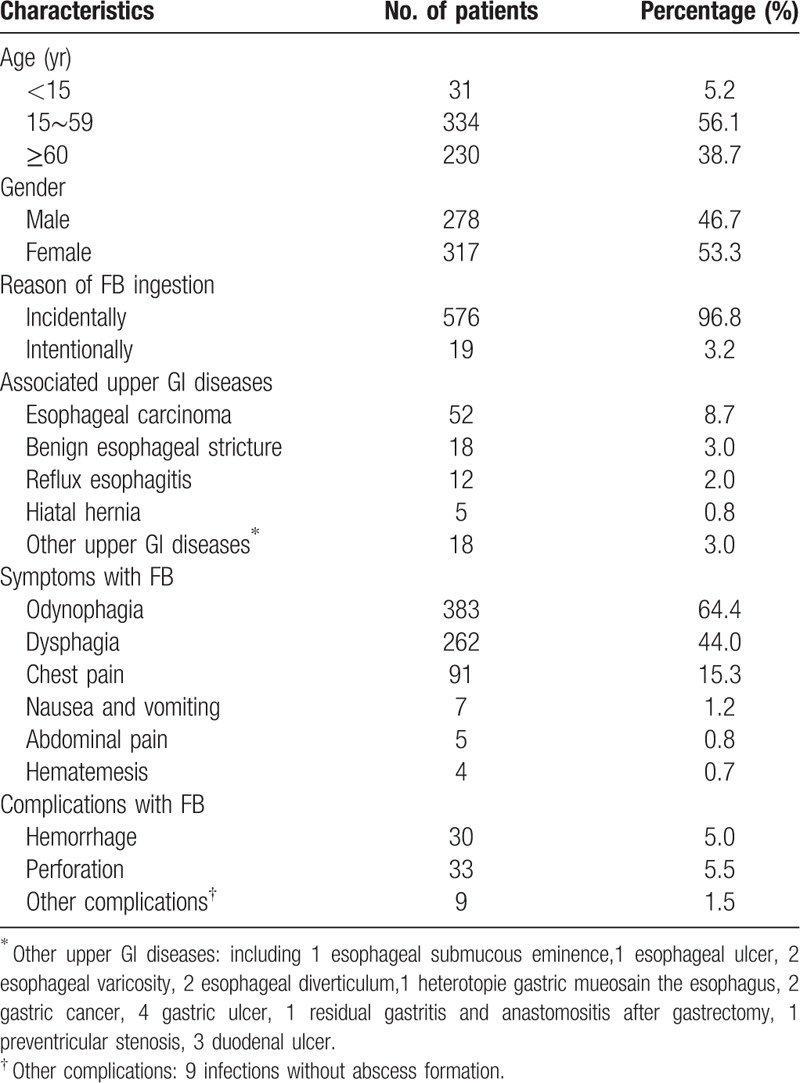
Characteristics of 595 patients with suspected FB ingestion.

### 3.2 Endoscopic outcomes and characteristics of foreign bodies

As the symptoms displayed by patients were often urgent and painful, the time interval from patient complaint to endoscopy management was recorded. Of the patients, 285 (50.8%) were treated with endoscopy management within 12 hours; 193 (34.4%), from 12 to 24 hours; 40 (7.1%), from 24 to 48 hours; 12 (2.2%), from 48 to 72 hours; and 31 (5.5%), longer than 72 hours.

The FBs in 426 patients were anatomically lodged at the proximal segment of the esophagus (75.9%), followed by the middle (15.2%) and distal segments (8.9%). According to the size of the FBs, 311 FBs (57.5%) were shorter than 2.5 cm, and 230 were with size longer than 2.5 cm (26.0%) (Table 2). In addition, 20 patients lacked records of the length of the FBs because the detected FBs were food boluses either pushed into the stomach or destroyed by piecemeal extraction. The most common type of ingested FBs in the esophagus was sharp objects (75.9%), including fish bones (34.0%), chicken bones (22.1%), and fruit nuclei (17.1%). The other type was non-sharp objects, including food bolus (14.6%), and coins (4.3%) (Fig. S2). According to the emergent situation, 428 cases (71.9%) were removed with local oral anesthesia and the rest (28.1%) were removed under general anesthesia (Table 2 and Table 3).

**Table 2:**
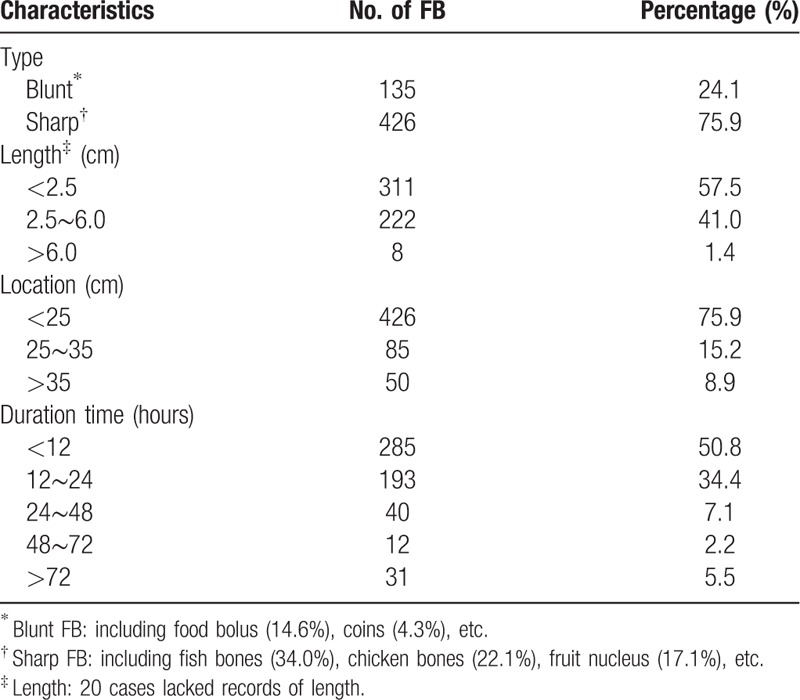
Characteristics of 561 visible FB under endoscopy.

**Table 3:**
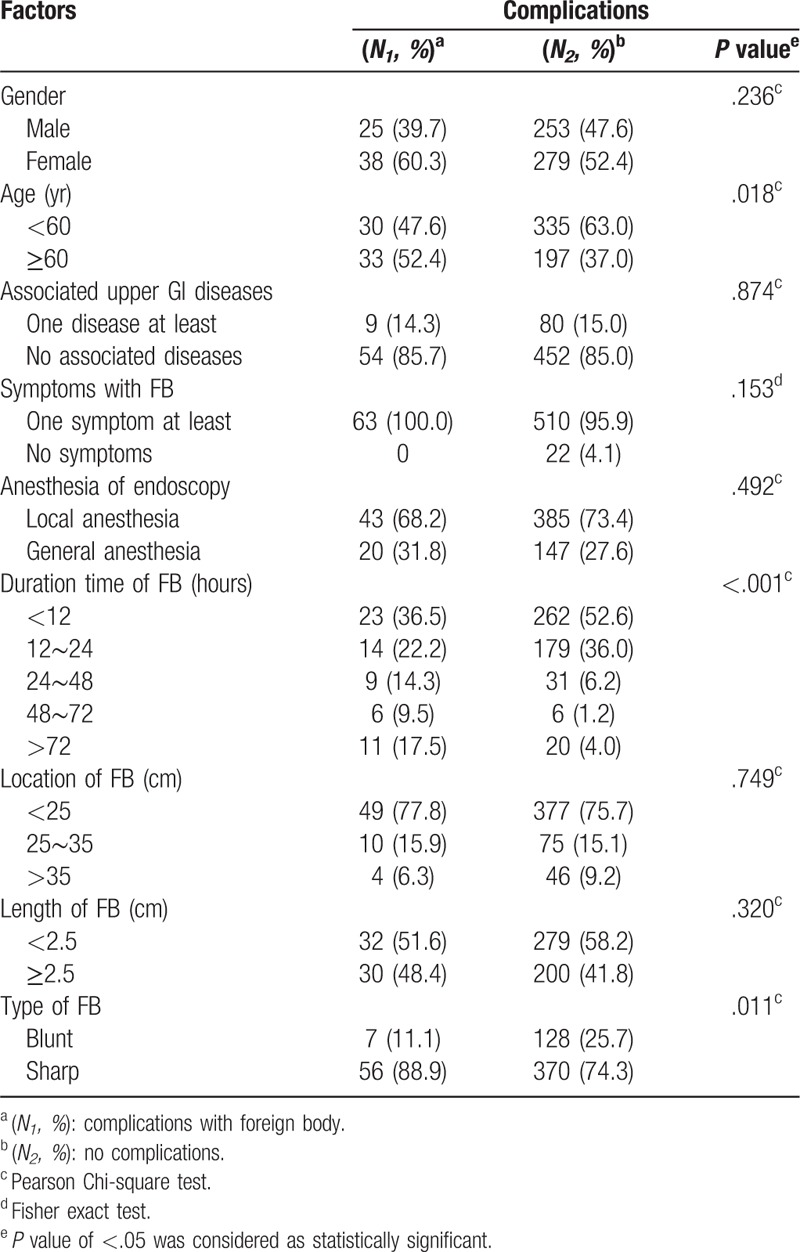
Univariate analysis of risk factor for complications.

**Table 4:**
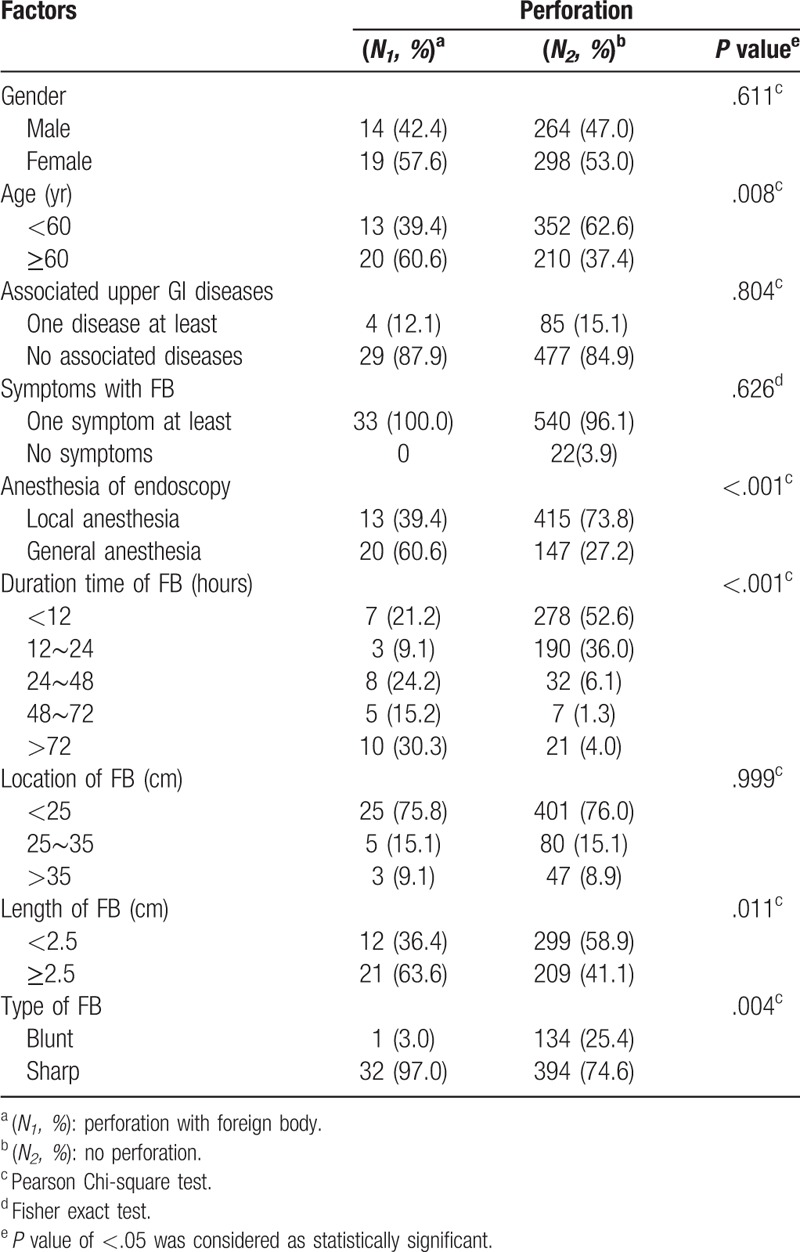
Univariate analysis of risk factor for perforation.

In the present study, the successful removal rate of FBs by endoscopy was 94.3%. In 17 cases, the FB was pushed into the stomach. The remaining 15 cases that were not removed were mainly associated with sharp FBs and local oral anesthesia. The univariate and logistic regression analyses indicated that general anesthesia could increase the successful removal rate by endoscopy (OR, 12.10; 95% CI, 1.56–93.80; *P* = .017) (Tables 5 and 6). Two patients required surgery, 1 patient had esophageal stenting for esophageal stricture, 6 patients were hospitalized to continue chemotherapy or radiotherapy, 1 patient died from multiple organ dysfunction syndrome, and the remaining patients successfully received with conservative treatment and were discharged.

**Table 5:**
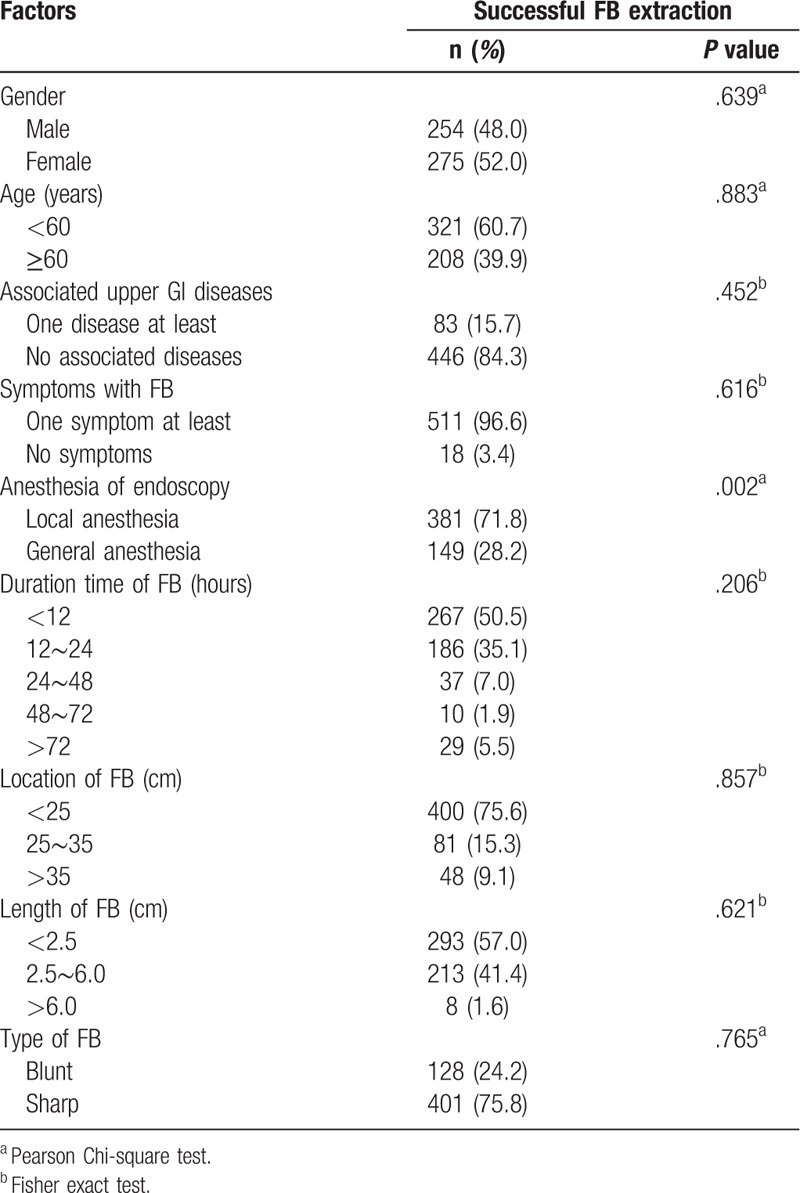
Univariate analysis of risk factor for successful esophagoscopic FB extraction rate.

**Table 6:**
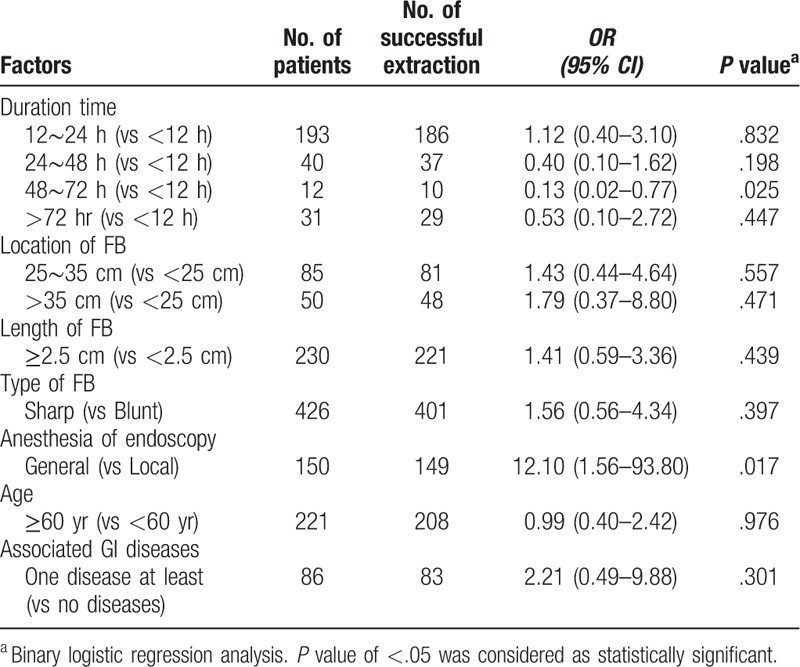
Multivariate analysis of risk factors for successful esophagoscopic FB extraction rate.

### 3.3 Complications and its attributing factors

**Figure 1:**
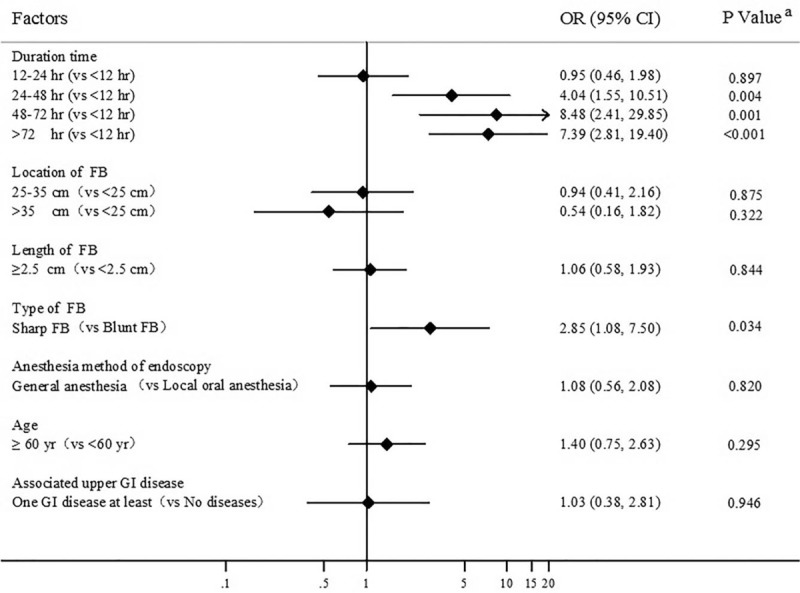
Multivariate analysis of risk factors of complications. FB = foreign body, GI = Gastrointestinal tract. ^a^Binary logistic regression analysis. A *P* value of <.05 was considered statistically significant.

Complications were found in 30 patients with hemorrhage (5.0%), 33 patients with perforation (5.5%), and 9 patients with infection (Table 1). Other common lesions included mucosal erosion (10.8%), laceration (9.1%), and ulcer (6.6%). According to the univariate analysis, among the risk factors attributed to the above-mentioned complications, older age (*P* = .018), long retention time (*P* < .001), and sharp FBs (*P* = .011) significantly increased the occurrence of complications (Table 3). Logistic regression analysis further indicated that sharp FBs (OR, 2.85; 95% CI, 1.08–7.50; *P* = .034) were associated with high risk of complications. Moreover, longer retention time significantly increased the occurrence of complications (*P* < .001). The risk increased dramatically by 4.04- and 8.48- fold with when the lodging time longer than 24 and 48 hours, respectively, as compared with less than 12 hours (Fig. 1).

### 3.4 Esophageal perforation and its attributing risk factors

**Figure 2:**
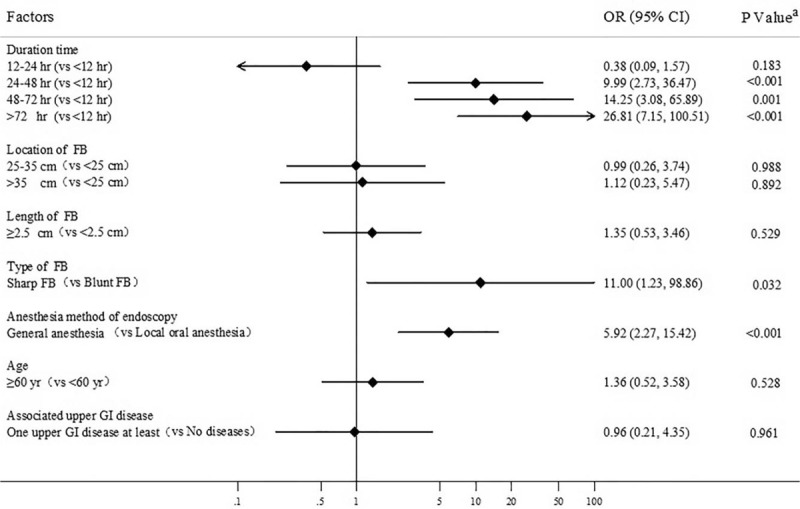
Multivariate analysis of risk factors of perforation. FB = foreign body, GI = Gastrointestinal tract. ^a^Binary logistic regression analysis. A *P* value of <.05 was considered statistically significant.

Esophageal perforation was one of the most severe complications. The risk of perforation increased by 9.99- and 26.81- fold when the FBs were retained for over 24 and 72 hours, respectively, as compared with less than 12 hours (Fig. 2). The univariate and multivariate analyses indicated that general anesthesia (OR, 5.92; 95% CI, 2.27–15.42; *P* < .001) and sharp objects (OR, 11.00; 95% CI, 1.23–98.86, *P* = .032) significantly increased the risk of perforation. However, no significant correlations were found between the incidence of perforation and the different FB locations or lengths of FBs (*P* > .05).

## 4 Discussion

Endoscopy remains a main intervention tool for removing impacted objects.^[5,16]^ However, large-scale populations with prospective and multi-center studies regarding the endoscopic management of FB are still lacking in China. In this study, the most common type of FBs was fish bones, subsequently followed by chicken bones, fruit nuclei, and food bolus. This was similar to that shown in previous reports in China or other Asian countries in adults.^[4,6]^ In Western countries, food bolus was the major type of impacted FB in adults.^[1,16]^ This variation might be correlated with the geographical and cultural differences in dietary habits.^[3,4]^ In addition, patients had varied GI diseases and complications among the different countries. For example, in Western countries, approximately 30% of patients had upper GI diseases, including eosinophilic esophagitis, esophageal carcinoma, esophageal stricture, and hiatus hernia.^[1,17]^ It was concluded that these patients had a higher risk of food impaction.^[18]^ However, in our study, 15% of the patients had upper GI diseases, mainly including esophageal carcinoma and stricture. The incidence of diseases might be correlated with the lower percentage of food bolus impaction in our study.

In emergent FB impaction cases, patient outcome is often determined by clinical complications. As previously mentioned, the FB associated complications include hemorrhage, perforation, obstruction, severe mucosal laceration and abscess formation.^[19]^ We found that 10.5% of the patients had complications, including hemorrhage (5.0%), perforation and infection (5.5%). Previous studies reported that the incidence of FB-related complications was 3% to 20%.^[20]^ Furthermore, we found that long retention time and sharp objects were the attributing risk factors of the aforementioned complications. The complication rate was increased by 4.04- fold when endoscopic retrieval was performed after impacted for over 24 hours as compared with within 12 hours. This finding has been demonstrated by other studies. A study of 401 cases in Hong Kong summarized that FBs trapped in the proximal esophagus or retained for over 48 hours had increased the risk of complications.^[12]^ In the United States, a study of 262 cases in a lower socioeconomic population found that 7.0% of the patients had complications, including perforation and bleeding, which were associated with the retention time and type of FBs.^[16]^ Esophageal FBs impacted for more than 24 hours might even have a 14.1-fold increase in the risk of complications.^[9]^ Apart from the retention time, the sharp feature of FBs could be another important attributing factor for complications. Lately, the ASGE^[5]^ and ESGE^[8]^ recommended therapeutic endoscopy for all cases of esophageal FBs within 24 hours after ingestion, especially for sharp-pointed objects within 6 hours. Here, we implicated that esophageal FBs, especially sharp objects, should be removed within 24 hours to decrease the incidence of devastating complications, which might be applicable to Asian populations.

As one of the most severe complications, perforation was found in 5.5% of our patients. This was strongly associated with the long retention time and sharp feature of the FBs, in accordance with previous reports.^[13,16]^ Previous studies indicated that objects longer than 6 cm increased the risk of perforation.^[11]^ However, this did not correlate with the anatomical locations and lengths of the FBs in our study. Hence, more investigations regarding sharp or long FBs are needed.

In addition to the above-mentioned factors, we found that general anesthesia prior to endoscopy increased the risk of perforation, although it might increase the successful removal rate by endoscopy. Owing to its favorable effects on reducing procedural pain and patient comfort, general anesthesia using propofol has been a mainstay approach in endoscopy,^[21]^ especially for some interventional procedures.^[22]^ However, adding general anesthesia to endoscopy is still controversial.^[23]^ In the context of esophageal FB impaction, general anesthesia did not significantly lower the complication rate as compared to topical pharyngeal anesthesia.^[14]^ Our finding of increased rate of perforation might be correlated with delayed treatment timing, the severity of patient, procedural complexity, and endoscopist experience. Recently, the ASGE suggested that endotracheal intubation was required for patients with objects that are difficult to be removed, patients with multiple objects, or while using rigid esophagogastroduodenoscopy. Under some circumstances, general anesthesia and endotracheal intubation might be needed, such as in children, psychiatric patients, incarcerated individuals or patients who cannot stand throughout the procedure.^[5]^ In order to avoid the risk of aspiration with blunting of airway protective reflexes, patients undergoing sedation have to largely empty the stomach before the whole procedure.^[24]^ It may delay the timing of manipulation and might increase the risk of perforation specifically with sharp FB impaction. And after deeply sedated, patients are unable to provide painful feedback to the endoscopist regarding gastrointestinal-wall excessive pressure from endoscopy no matter whether perforation is happening or not. This is accordant to the finding from a prospective cohort study claiming a higher perforation rate in colonoscopies with anesthesia services.^[25]^

FB ingestion is a global common medical emergency. FB-related complications significantly determined clinical patient outcomes. Our study provides reference for endoscopic management of FB ingestion in the esophagus. We found sharp objects, the most frequently ingested FBs in the esophagus in China, were associated with high risk of complications. Delayed endoscopic retrieval for over 24 hours increased the complication rate when compared with within 12 hours. As long retention time and sharp objects were the attributing risk factors of complications, we recommend that sharp FBs should be removed within 24 hours after ingestion. There are several limitations and explanations. First, due to the lack of anesthesiologist in the emergency endoscopy, general anesthesia only accounted for 28.1%. This is consistent with the status all across the country but is quite different from Western countries. Under this special circumstance, the frequency of general anesthesia is positively correlated with the FB retention time, which is attributable risk factor to complications. Second, there are no strict requirements on the standardize endoscopist qualification. Hence, further investigations about the safety of endoscopic removal of FBs in the presence or absence of sedation are warranted.

## 5 Conclusion

Early management and risk stratification is the key for the emergency of FB ingestion in the esophagus. Our study highlighted that the most common FBs were sharp objects. Moreover, sharp objects and long retention time strongly increased the incidence of complications, especially perforation. These implicate that sharp FBs should be removed within 24 hours after ingestion.

## Author contributions

**Conceptualization:** Bili He, Zhenzhai Cai, Bin Ye, Lei Xu, Jiang Liu, Jin Ding, Zhongfa Zheng, Jianhua Duan.

**Data curation:** Jingjing Yuan, Mengjie Ma, Bili He, Zhenzhai Cai.

**Formal analysis:** Jingjing Yuan, Mengjie Ma, Yang Guo, Liangjing Wang.

**Investigation:** Jingjing Yuan, Mengjie Ma, Liangjing Wang.

**Methodology:** Jingjing Yuan, Yang Guo, Zhenzhai Cai, Bin Ye, Lei Xu, Jiang Liu, Jin Ding, Zhongfa Zheng, Jianhua Duan, Liangjing Wang.

**Project administration:** Liangjing Wang.

**Resources:** Bili He, Zhenzhai Cai, Bin Ye, Lei Xu, Jiang Liu, Jin Ding, Zhongfa Zheng, Jianhua Duan, Liangjing Wang.

**Software:** Jingjing Yuan.

**Supervision:** Jingjing Yuan, Bili He, Bin Ye, Liangjing Wang.

**Writing – original draft:** Jingjing Yuan, Mengjie Ma.

**Writing – review & editing:** Jingjing Yuan, Mengjie Ma, Yang Guo, Liangjing Wang.

## Supplementary Material

Supplemental Digital Content

## References

[1] Mosca Manes G Martino R Endoscopic management of foreign bodies in the upper gastrointestinal tract: report on a series of 414 adult patients. Endoscopy 2001;33:692–6. 11490386 10.1055/s-2001-16212

[2] Emara MH Darwiesh EM Refaey MM Endoscopic removal of foreign bodies from the upper gastrointestinal tract: 5-year experience. Clin Exp Gastroenterol 2014;7:249–53. 25053889 10.2147/CEG.S63274 PMC4105424

[3] Li ZS Sun ZX Zou DW Endoscopic management of foreign bodies in the upper-GI tract: experience with 1088 cases in China. Gastrointest Endosc 2006;64:485–92. 16996336 10.1016/j.gie.2006.01.059

[4] Zhang S Cui Y Gong X Endoscopic management of foreign bodies in the upper gastrointestinal tract in South China: a retrospective study of 561 cases. Dig Dis Sci 2010;55:1305–12. 19655249 10.1007/s10620-009-0900-7

[5] Committee ASoP Ikenberry SO Jue TL Management of ingested foreign bodies and food impactions. Gastrointest Endosc 2011;73:1085–91. 21628009 10.1016/j.gie.2010.11.010

[6] Park JH Park CH Park JH Review of 209 cases of foreign bodies in the upper gastrointestinal tract and clinical factors for successful endoscopic removal. Korean J Gastroenterol 2004;43:226–33. 15100486

[7] Kramer RE Lerner DG Lin T Management of ingested foreign bodies in children: a clinical report of the NASPGHAN Endoscopy Committee. J Pediatr Gastroenterol Nutr 2015;60:562–74. 25611037 10.1097/MPG.0000000000000729

[8] Birk M Bauerfeind P Deprez PH Removal of foreign bodies in the upper gastrointestinal tract in adults: European Society of Gastrointestinal Endoscopy (ESGE) Clinical Guideline. Endoscopy 2016;48:489–96. 26862844 10.1055/s-0042-100456

[9] Loh KS Tan LK Smith JD Complications of foreign bodies in the esophagus. Otolaryngol Head Neck Surg 2000;123:613–6. 11077351 10.1067/mhn.2000.110616

[10] Tokar B Cevik AA Ilhan H Ingested gastrointestinal foreign bodies: predisposing factors for complications in children having surgical or endoscopic removal. Pediatr Surg Int 2007;23:135–9. 17043873 10.1007/s00383-006-1819-0

[11] Mendelson AH Small AJ Agarwalla A Esophageal anastomotic strictures: outcomes of endoscopic dilation, risk of recurrence and refractory stenosis, and effect of foreign body removal. Clin Gastroenterol Hepatol 2015;13:263–71. e261. 25019695 10.1016/j.cgh.2014.07.010 PMC4289652

[12] Lai AT Chow TL Lee DT Risk factors predicting the development of complications after foreign body ingestion. Br J Surg 2003;90:1531–5. 14648732 10.1002/bjs.4356

[13] Vidarsdottir H Blondal S Alfredsson H Oesophageal perforations in Iceland: a whole population study on incidence, aetiology and surgical outcome. Thorac Cardiovasc Surg 2010;58:476–80. 21110271 10.1055/s-0030-1250347

[14] Geng C Li X Luo R Endoscopic management of foreign bodies in the upper gastrointestinal tract: a retrospective study of 1294 cases. Scand J Gastroenterol 2017;52:1286–91. 28691540 10.1080/00365521.2017.1350284

[15] Cheng W Tam PK Foreign-body ingestion in children: experience with 1,265 cases. J Pediatr Surg 1999;34:1472–6. 10549750 10.1016/s0022-3468(99)90106-9

[16] Palta R Sahota A Bemarki A Foreign-body ingestion: characteristics and outcomes in a lower socioeconomic population with predominantly intentional ingestion. Gastrointest Endosc 2009;69 (3 Pt 1):426–33. 19019363 10.1016/j.gie.2008.05.072

[17] Dray X Cattan P Foreign bodies and caustic lesions. Best Pract Res Clin Gastroenterol 2013;27:679–89. 24160927 10.1016/j.bpg.2013.08.009

[18] Longstreth GF Longstreth KJ Yao JF Esophageal food impaction: Epidemiology and therapy. A retrospective, observational study. Gastrointest Endosc 2001;53:193–8. 11174291 10.1067/mge.2001.112709

[19] Silva RG Ahluwalia JP Asymptomatic esophageal perforation after foreign body ingestion. Gastrointest Endosc 2005;61:615–9. 15812424 10.1016/s0016-5107(05)00081-7

[20] Gmeiner D von Rahden BH Meco C Flexible versus rigid endoscopy for treatment of foreign body impaction in the esophagus. Surg Endosc 2007;21:2026–9. 17393244 10.1007/s00464-007-9252-6

[21] Cohen LB Wecsler JS Gaetano JN Endoscopic sedation in the United States: results from a nationwide survey. Am J Gastroenterol 2006;101:967–74. 16573781 10.1111/j.1572-0241.2006.00500.x

[22] Shin S Park CH Kim HJ Patient satisfaction after endoscopic submucosal dissection under propofol-based sedation: a small premedication makes all the difference. Surg Endosc 2017;31:2636–44. 27743126 10.1007/s00464-016-5276-0

[23] Birk J Bath RK Is the anesthesiologist necessary in the endoscopy suite? A review of patients, payers and safety. Expert Rev Gastroenterol Hepatol 2015;9:883–5. 25979248 10.1586/17474124.2015.1041508

[24] Committee ASoP Early DS Lightdale JR Guidelines for sedation and anesthesia in GI endoscopy. Gastrointest Endosc 2018;87:327–37. 29306520 10.1016/j.gie.2017.07.018

[25] Wernli KJ Brenner AT Rutter CM Risks associated with anesthesia services during colonoscopy. Gastroenterology 2016;150:888–94. quiz e818. 26709032 10.1053/j.gastro.2015.12.018 PMC4887133

