# Supplementary material for: Delayed endoscopic removal of sharp foreign body in the esophagus increased clinical complications: An experience from multiple centers in China
Source: Medicine (Baltimore). 2019 Jun 28;98(26):e16146. doi: 10.1097/MD.0000000000016146 (PMC6617444; doi:10.1097/MD.0000000000016146)

**Figure S1.**  Symptoms in 595 patients with foreign body ingestion. Nearly 10% of the patients had more than one symptom.


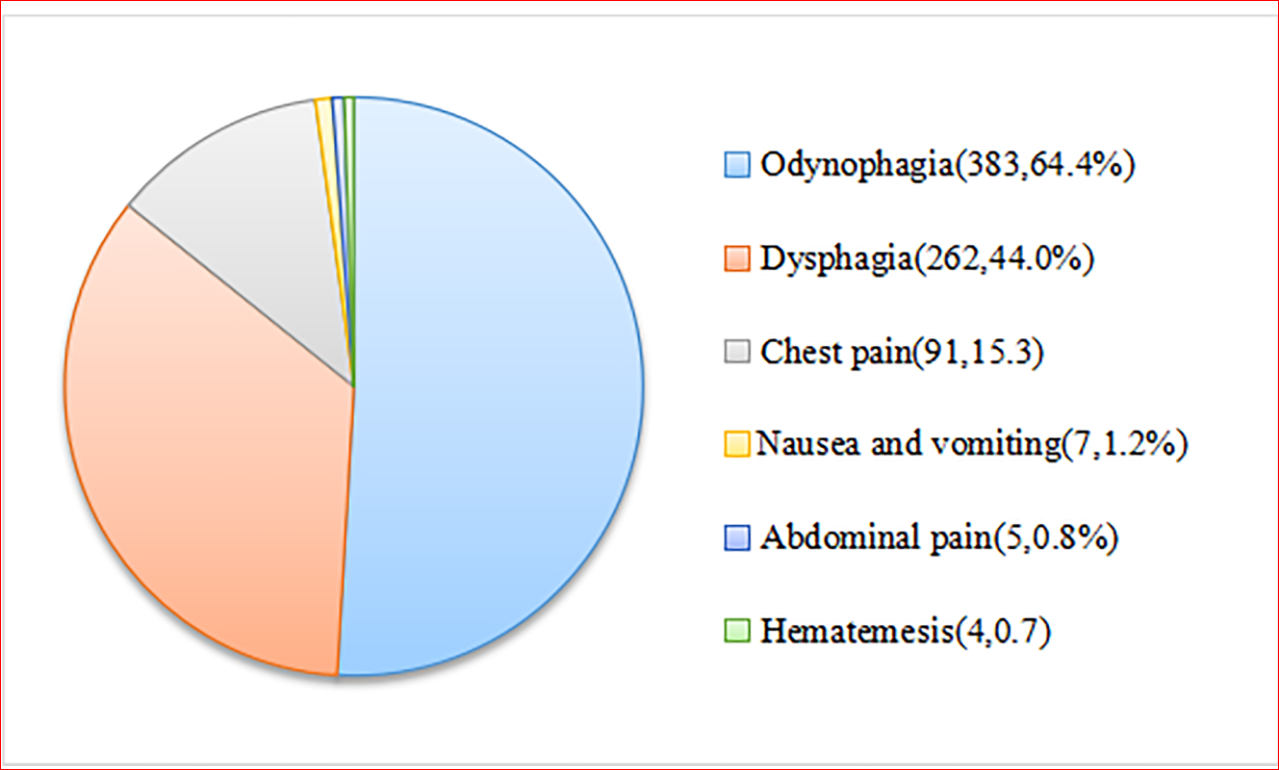


**Figure S2.** Type of the 561 visible foreign bodies under endoscopy. Other types of foreign body, including metal rods, toothpicks, pills, buttons, rings, and bottle caps.


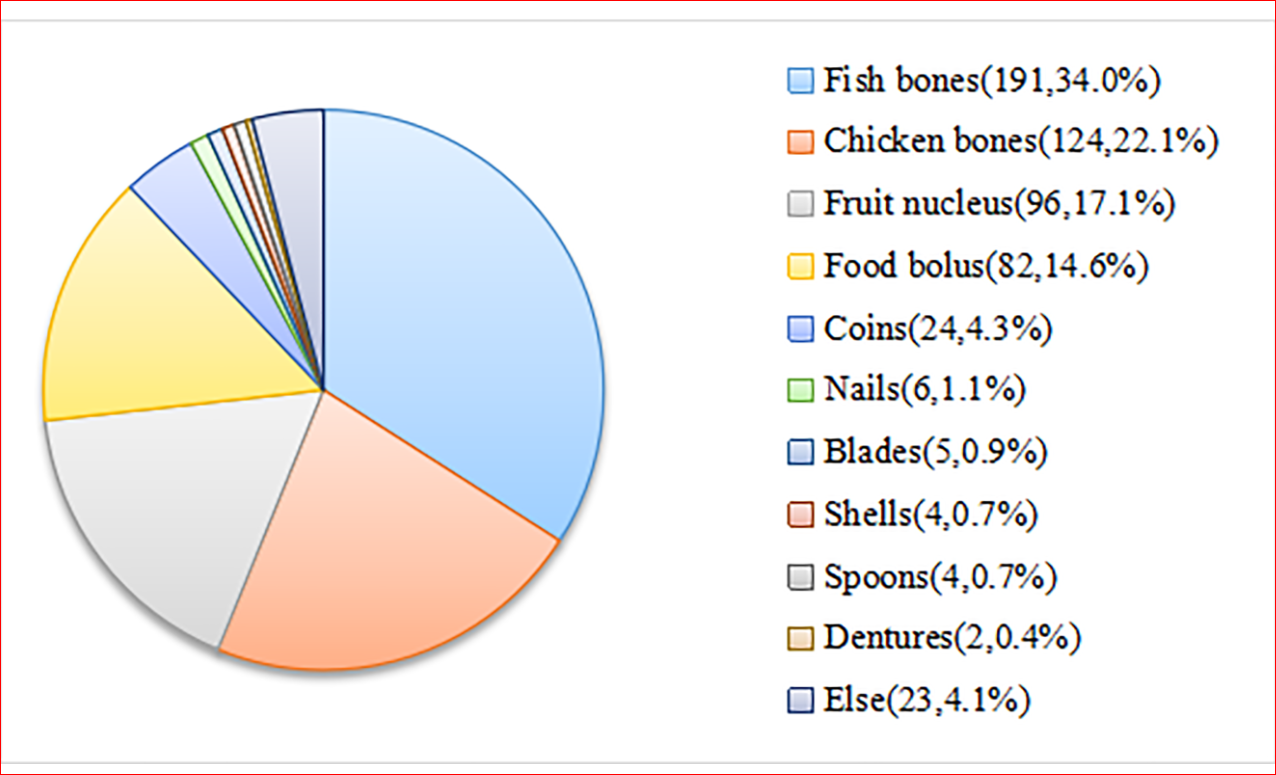

Supplement: Supplemental Digital Content [file medi-98-e16146-s001.doc]
